# Virtual Reality for Coronary Angiography: A Step Forward in Patient-Centered Anxiety Management

**DOI:** 10.1016/j.jacadv.2025.102327

**Published:** 2025-11-07

**Authors:** Ioannis Skalidis, Philippe Garot, Thomas Hovasse, Mariama Akodad, Stephane Cook

**Affiliations:** a Institut Cardiovasculaire Paris-Sud, Hôpital Jacques Cartier, Massy, France; b Interventional Cardiology Department, University and Hospital Fribourg, Fribourg, Switzerland

Preprocedural anxiety remains a pervasive challenge in cardiovascular interventions, particularly in patients undergoing invasive coronary angiography (ICA), where psychological distress can adversely influence both patient experience and clinical outcomes. In this context, nonpharmacological approaches such as immersive virtual reality (VR) have emerged as promising adjuncts to standard anxiolytic care. The recent randomized controlled trial by Groenveld et al.^1^ offers valuable insights into the role of VR therapy in mitigating preprocedural anxiety among patients undergoing ICA. The authors should be commended for executing a methodologically robust investigation, featuring both intention-to-treat and per-protocol analyses, and for targeting a clinically relevant and often under-recognized issue.

A notable strength lies in the study’s pragmatic approach, simulating a real-world environment with a broad patient population, including those with non-ST-segment elevation acute coronary syndrome, coronary function testing, and elective ICA. Particularly intriguing is the finding that after adjustment for baseline anxiety, VR therapy yielded a statistically and clinically significant reduction in preprocedural anxiety, with the greatest effect observed in patients with non-ST-segment elevation acute coronary syndrome. This subgroup may represent a population with heightened baseline uncertainty and limited preparatory time, thereby magnifying the anxiolytic benefit of immersive distraction. This observation warrants further investigation into tailoring VR interventions based on procedural urgency and patient expectations.^2^

Secondly, although the absence of corresponding effects on physiological markers such as heart rate variability and respiratory rate may reflect pharmacological confounders (eg, beta-blocker use), it raises the question of whether self-reported anxiety scales alone suffice to capture the multidimensional stress response. Future studies incorporating biomarkers of sympathetic activation, cortisol levels, or neuroimaging data could deepen mechanistic understanding and validate the subjective outcomes.

Lastly, the absence of intraprocedural VR continuation, except in select patient-initiated cases, may have limited the potential benefit. Given the growing interest in conscious sedation alternatives during interventional procedures, would the authors consider evaluating extended VR exposure throughout the angiographic procedure in a subgroup not requiring continuous verbal interaction? Such a design may delineate the maximum achievable effect of VR in cardiac interventions and expand its applicability beyond preprocedural use.^3^

Overall, the VR InCard Trial enriches the evidence base for digital interventions in cardiovascular care and sets the stage for refined, indication-specific, and potentially intraprocedural applications of VR therapy in anxiety-sensitive settings.
